# Transcriptome analysis identifies metallothionein as biomarkers to predict recurrence in hepatocellular cacinoma

**DOI:** 10.1002/mgg3.693

**Published:** 2019-05-06

**Authors:** Sufang Wang, Michael Gribskov

**Affiliations:** ^1^ School of Life Sciences Northwestern Polytechnical University Xi'an Shaanxi China; ^2^ Center of Special Environmental Biomechanics & Biomedical Engineering Northwestern Polytechnical University Xi'an Shaanxi China; ^3^ Department of Biological Sciences Purdue University West Lafayette Indiana USA; ^4^ Department of Computer Sciences Purdue University West Lafayette Indiana USA

**Keywords:** biomarker, de novo transcriptome assembly, hepatocellular carcinoma, metallothionein, recurrence

## Abstract

**Background:**

Liver cancer is the fifth most common cancer, and hepatocellular carcinoma (HCC) is the major liver tumor type seen in adults. HCC is usually caused by chronic liver disease such as hepatitis B virus or hepatitis C virus infection. One of the promising treatments for HCC is liver transplantation, in which a diseased liver is replaced with a healthy liver from another person. However, recurrence of HCC after surgery is a significant problem. Therefore, it is important to discover reliable cellular biomarkers that can predict recurrence in HCC.

**Methods:**

We analyzed previously published HCC RNA‐Seq data that includes 21 paired tumor and normal samples, in which nine tumors were recurrent after orthotopic liver transplantation and 12 were nonrecurrent tumors with their paired normal samples. We used both the reference genome and de novo transcriptome assembly based analyses to identify differentially expressed genes (DEG) and used RandomForest to discover biomarkers.

**Results:**

We obtained 398 DEG using the Reference approach and 412 DEG using de novo assembly approach. Among these DEG, 258 genes were identified by both approaches. We further identified 30 biomarkers that could predict the recurrence. We used another independent HCC study that includes 50 patients normal and tumor samples. By using these 30 biomarkers, the prediction accuracy was 100% for normal condition and 98% for tumor condition. A group of Metallothionein was specifically discovered as biomarkers in both reference and de novo assembly approaches.

**Conclusion:**

We identified a group of Metallothionein genes as biomarkers to predict recurrence. The metallothionein genes were all down‐regulated in tumor samples, suggesting that low metallothionein expression may be a promoter of tumor growth. In addition, using de novo assembly identified some unique biomarkers, further confirmed the necessity of conducting a de novo assembly in human cancer study.

## 1 INTRODUCTION

Liver cancer is the fifth most common cancer, and the third leading cause of cancer‐related death worldwide. Hepatocellular carcinoma (HCC) is the major liver tumor type seen in adults (Bosch, Ribes, Díaz, & Cléries, 2004; Shibata & Aburatani, 2014; Thomas et al., 2010). HCC is usually caused by chronic liver disease such as hepatitis B virus (HBV) or hepatitis C virus (HCV) infection, which accounts for 75%–80% of the cases (Arzumanyan, Reis, & Feitelson, 2013; Bosch et al., 2004). Abuse of alcohol and exposure to aflatoxin are also risk factors for HCC.

The pathogenic mechanisms of hepatitis B or C associated HCC have been heavily investigated. Alterations in the activities and expression levels of several signaling pathways have been identified. For example, inactivation of tumor suppressor genes *p53* (Christofori, Naik, & Douglas, 1995), *RAS* (Oishi et al., 2007), *PI3K* (Zender et al., 2008), overexpression of β‐catenin in the Wnt signaling pathways (Edamoto et al., 2003; Peng et al., 2004), overexpression of epidermal growth factor receptor family members (Blivet‐Van Eggelpoël et al., 2012; Ito et al., 2001), overexpression of *MET* and its ligand hepatocyte growth factor (Daveau et al., 2003) and overexpression of insulin‐like growth factor (Sedlaczek, Hasilik, Neuhaus, Schuppan, & Herbst, 2003). In addition, methylation of cancer relevant genes have been also identified (Kubo et al., 2004; Lee et al., 2003; Liew et al., 1999; Matsuda, Ichida, Matsuzawa, Sugimura, & Asakura, 1999; Murata et al., 2004; C. Wong, Lee, Ching, Jin, & Ng, 2003; I. H. N. Wong et al., 1999), including *APC*,* p16*, E‐cadherin, *GSTP1*,* COX2*, apoptosis‐associated speck‐like protein (*ASC*) and deleted in liver cancer 1, and allelic gains or losses on chromosomes (Kuroki et al., 1995; Maggioni, 2000; Wilkens et al., 2001). However, due to the heterogeneity of HCC, it is not yet clear what early biomarkers could be used for detection of HBV or HCV‐mediated HCC (Arzumanyan et al., 2013).

The treatment for HCC includes liver resection, liver transplantation, chemotherapy, and radiation. Liver transplantation is one of the promising treatments, in which a diseased liver is replaced with a healthy liver from another person. However, recurrence of HCC after surgery is a significant problem. It has been reported that the recurrence after liver transplantation ranges from 6% to 40% (Cheng et al., 2011; Marsh et al., 1997; Shimoda et al., 2004). Although, many studies have attempted to identify biomarkers, in order to predict recurrence in patients with HCC, the early detection of recurrence still remains challenge. The current biomarkers for HCC are mostly serum markers, which show low sensitivity (Tsuchiya et al., 2015). Three recent studies identified some novel markers. One was using DNA markers from urine, but the study was based on only 10 individual patients and did not show wide applicability (Hann et al., 2017); a meta‐analysis showed a possibility of using circRNA as biomarkers, but they did not talk about the recurrence problem in HCC (M. Wang et al., 2018); marker for the prediction of sorafenib response has been proposed, but its relevance to the recurrence problem is unclear(Kim et al., 2018). Therefore, it is important to discover reliable cellular biomarkers that can predict recurrence in HCC.

Next‐generation sequencing, which identifies genomic alterations and somatic mutations at the nucleotide base level, is providing insights into the etiology of cancer and corresponding diagnostics (Chin et al., 2012; Davey et al., 2011; Meyerson, Gabriel, & Getz, 2010; Schuster, 2008). Scientists have started to sequence patients’ DNA or mRNA to obtain their genome or transcriptome, but gene expression is usually measured based on the annotation of the human reference genome. Recently, it has been suggested that de novo assembly is valuable even when a reference genome is available (S. Wang & Gribskov, 2017). Although the human reference genome is available, in the case of tumors, where mutation and chromosomal rearrangement may have altered gene/transcript structure, incorporation of de novo assembly is even more important.

In this project, we analyzed previously published HCC RNA‐Seq data (Xue et al., 2015) that includes 21 paired tumor and normal samples, in which nine tumors were recurrent after orthotopic liver transplantation and 12 were nonrecurrent tumors with their paired normal samples. In this study, we compared the results of reference and de novo transcriptome assembly based analyses, in order to identify biomarkers that predict recurrence of tumors in HCC.

## 2 MATERIALS AND METHODS

### 2.1 Data description

The RNA‐seq data were directly downloaded from the NCBI sequence read archive (SRP040998). There were nine recurrent tumor with paired adjacent normal samples, and 12 nonrecurrent tumor with paired adjacent normal samples. In total, there were 9 × 2 + 12 × 2 = 42 samples. Details of library construction and patient information are described in Xue et al. (2015).

### 2.2 Quality control of raw data

Adapter sequences and low‐quality portions of reads were removed using Trimmomatic (version 0.32) (Bolger, Lohse, & Usadel, 2014). Adapters and low‐quality read regions with average quality below 13 (phred score) over a four base window were removed. Low‐quality sequences at the 5′ and 3′ end, with quality score <10 were also removed. Only reads with a trimmed length over 30 bases were used in further analysis. The number of paired‐end reads in each sample is shown in Table S1.

### 2.3 Transcriptome assembly of cleaned data

We pooled all left cleaned reads, right cleaned reads, and unpaired cleaned reads from all 42 samples together for de novo transcriptome assembly. Cleaned reads were assembled using Trinity (Grabherr et al., 2011) (version 2.0.6) with the parameters recommended by the authors.

### 2.4 Alignment and quantification

Bowtie2 was used to align cleaned reads to both human reference genome (GRCH38) and de novo transcriptome assembly. Then RSEM (version 1.2.30) program (Li & Dewey, 2011; Li, Ruotti, Stewart, Thomson, & Dewey, 2010) was used to quantify gene expression level.

### 2.5 Differential expression gene analysis

The DeSeq2 package (Love, Huber, & Anders, 2014) was used to determine differential expression. The integrated statistical model is:

$$Y_{\mathrm{ijk}}=\mu +\alpha_{i}+\beta_{j}+\varepsilon_{\mathrm{ijk}},$$

where *i* is group (recurrent or nonrecurrent), *j* is condition (normal or tumor), *k* is individual (patient). In this model, we integrated the sample type (tumor or normal) and recurrence type (yes or no), which identified genes that were both differentially expressed in these conditions. Only genes with observed counts >100 (summed over all conditions) were analyzed.

### 2.6 Blast search

We compared the de novo assembly to the human reference genome (GRCH38) using BlastN with default settings (Blast version 2.2.29+, National Center for Biotechnology Information, National Library of Medicine, National Institues of Health, Bethesda, MD, USA). We filtered hits by two criteria: identity score ≥95%; and aligned length ≥100 bases.

### 2.7 Biomarker identification and confirmation

The RandomForest package (Liaw & Wiener, 2002) was used to identify biomarkers from recurrent and nonrecurrent patients’ gene expression levels. Another independent data set was downloaded from the NCBI sequence read archive (SRP068976) for use as confirmation data, to predict the patient outcome using the biomarkers identified in the RandomForest analysis. The confirmation data included 50 patients paired normal and tumor RNA‐Seq data. Details of library construction and patient information are described in Liu et al. (2016).

## 3 RESULTS

### 3.1 De novo transcriptome assembly

We pooled all patient reads together to assemble the transcriptome using the Trinity program (*k‐mer* = 25). In total, there were 1,036,270 predicted transcripts in the assembly with minimum length 224 bp and maximum length 31,736 bases. Trinity labels predicted transcripts systematically with designated form such as TR_*i*_|*c*_*j*__*g*_*k*__*i*_*l*_ (e.g., TR_101_|*c*_0__*g*_2__*i*_2_), where *i*,* j*,* k,* and *l* are integers indicate the transcripts, component, group, and isoform, respectively. We determined that sequences with the same component (e.g., *c*_0_, *c*_1_) but different groups such as TR_1_|*c*_0__*g*_1_ and TR_1_|*c*_0__*g*_2_, usually match the same gene. Therefore, we used transcripts number for example, TR_1_ as the gene unit. The number of genes when pooled at this level in de novo transcriptome assembly was 797,713, substantially more than the number of genes annotated in the human reference genome. This is possibly due to two effects. (a) many of the predicted transcripts are similar or duplicated; (b) many of them are expressed at low levels which leads to incomplete transcripts.

### 3.2 Differentially expressed genes on recurrent/nonrecurrent tumor analysis

We analyzed nine recurrent tumors (after orthotopic liver transplantation) and 12 nonrecurrent tumors, each with a paired normal sample. We did analysis using two approaches, (a) all samples were analyzed with respect to the human Reference genome; (b) all samples were analyzed with respect to de novo assembly (Trinity). Thus, we obtained two gene expression profiles (one using the reference genome, the other using the assembly), and two differentially expressed genes (DEG) lists (see Materials and Methods2 for details).

**Figure 1:**
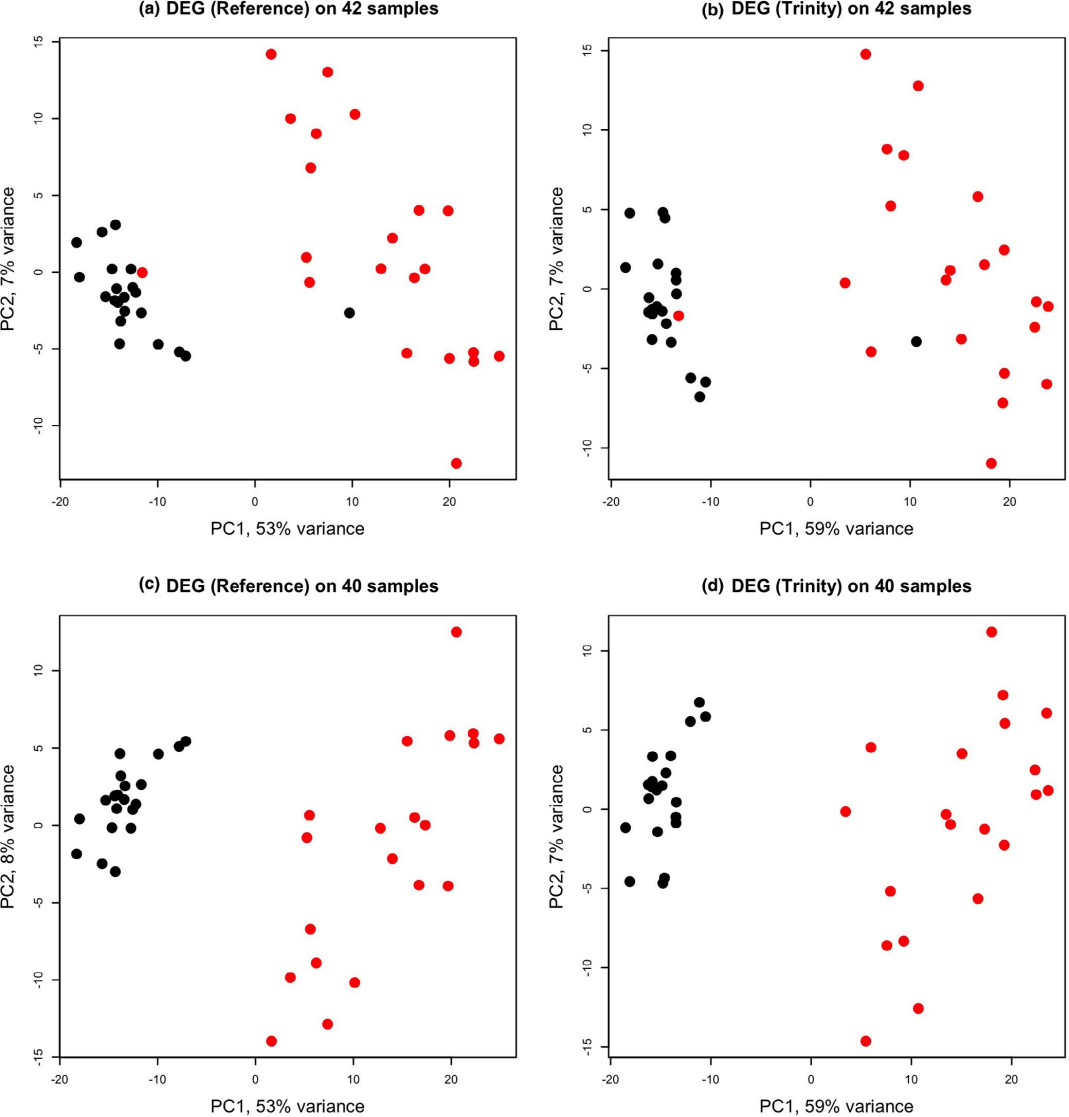
Principal component analyses on normal (black dots) and tumor (red dots) samples. (a) Principal component analyses for Reference approach. (b) Principal component analyses for de novo assembly (Trinity) approach. (c) Principal component analyses with one patient excluded in Reference approach. (d) Principal component analyses with one patient excluded in de novo assembly (Trinity) approach. DEG, differentially expressed gene

First, we used the gene expression level to do a principal component analysis. Gene counts were log_10_ transformed. All normal samples clustered closely, while the tumor samples were distributed widely in both the Reference and assembly cases (Figure 1a,b). However, data from one patient (both normal and tumor) showed very large reciprocal deviations from the expected clusters (Figure 1a,b), suggesting that the tumor and normal samples may be mislabeled. If this is the case, it would cause large errors in estimates of variance, suggesting this sample should be omitted. Without this patient, the paired normal and tumor samples, were better separated (Figure 1c,d). In order to gain more confidence in identifying DEG, we excluded this patient from all analysis.

DEG were identified using the DeSeq2 package with the integrated model where takes group (recurrent or nonrecurrent) and condition (normal or tumor) into account. The significance level was defined as a false discovery rate < 0.0001, and log_2_ fold‐change (log_2_FC) larger than ±3 (i.e., eightfold change). In total, we obtained 398 DEG using the Reference approach (Table S2) and 412 DEG using de novo assembly approach (Table S3). We further compared the DEG between these two methods and found that 258 DEG were identified by both approaches.

**Figure 2:**
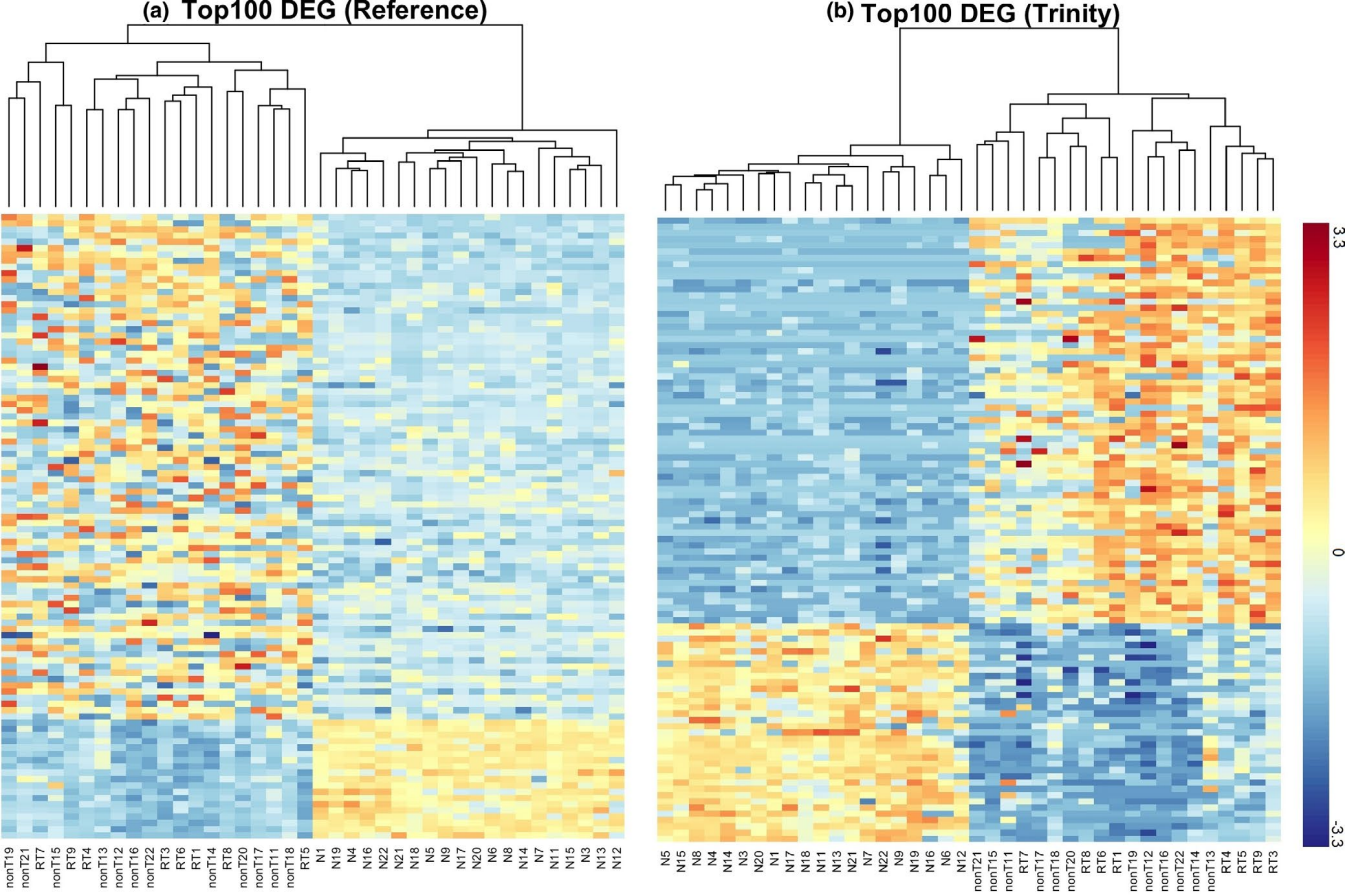
Heatmaps of top 100 DEG with largest fold change. The hierarchical clusters were based on *Z*‐score. (a) Heatmap of top 100 DEG identified using Reference approach (b) Heatmap of 100 DEG identified using de novo assembly (Trinity). DEG, differentially expressed gene

After identifying the DEG, we first checked DEG expression. In order to provide a more straightforward and detailed perspective on gene expression, up‐ and down‐regulated genes were displayed as a heatmap. We chose the top 100 DEG (for a better viewing) exhibiting the largest fold change and used hierarchical clustering with Euclidean distance and complete linkage method. Gene counts were log_10_ transformed and normalized as *Z*‐score. From the heatmap, there were clearly two clusters (Figure 2); all 20 normal samples were in one cluster and the 20 tumor samples in another cluster. The tumor and normal samples separated very well in both the Reference and de novo assembly (Figure 2), which gives the confidence that these DEG express differentially between tumor and normal samples.

### 3.3 Comparison with known cancer genes

In order to validate the involvement of the identified genes in cancer etiology, we compared the DEG to two cancer gene databases: tumor suppressor genes (Zhao, Kim, Mitra, Zhao, & Zhao, 2016; Zhao, Sun, & Zhao, 2013) and oncogenes (Y. Liu, Sun, & Zhao, 2017). We combined the two databases to produce a list of 1616 cancer oncogenes and tumor suppressor genes. We refer this combined database as known cancer genes and it serves as an internal positive control because we expect some of these known cancer genes to be identified in the analysis. We matched our DEG to this list and found 41 known cancer genes in Reference approach and 39 known cancer genes in de novo assembly approach. Twenty‐two known cancer genes were found using both methods (Table 1). Some of these cancer genes have previously identified in liver cancer. For example, the overexpression of insulin like growth factor 2 was found to be associated with HCC (Sedlaczek et al., 2003). This gives high confidence that the DEG represent genes involving in regulating pathways in HCC.

**Table 1:** Known cancer genes found using both methods

Name	Description	Reference		De novo assembly	
		log_2_FC	FDR	log_2_FC	FDR
*MT1G*	Metallothionein 1G	−4.1	3.96E‐18	−4.15	4.40E‐19
*MT1F*	Metallothionein 1F	−3.85	2.24E‐24	−4.84	1.47E‐22
*CXCL14*	C‐X‐C motif chemokine ligand 14	−3.61	1.00E‐11	−3.53	1.73E‐10
*RAB25*	Member of RAS oncogene family	−3.57	8.91E‐10	−4.27	2.12E‐09
*BMP10*	Bone morphogenetic protein 10	−3.31	4.66E‐09	−3.43	1.52E‐09
*SOX2*	SRY‐box 2	3.05	4.67E‐06	3.39	1.97E‐08
*CCNE1*	Cyclin E1	3.18	1.07E‐13	3.1	2.10E‐06
*CCNB1*	Cyclin B1	3.19	6.98E‐22	3.19	6.43E‐18
*MAFA*	MAF bZIP transcription factor A	3.27	4.21E‐06	3.66	4.61E‐09
*PTTG1*	Pituitary tumor‐transforming 1	3.27	3.83E‐18	3.74	7.13E‐10
*KIF14*	Kinesin family member 14	3.36	3.86E‐32	3.52	9.63E‐28
*CDK1*	Cyclin dependent kinase 1	3.38	2.18E‐22	3.33	1.94E‐19
*MYO18B*	Myosin XVIIIB	3.4	9.81E‐07	3.68	3.57E‐06
*CDKN3*	Cyclin dependent kinase inhibitor 3	3.64	3.63E‐23	3.43	6.51E‐19
*E2F1*	E2F transcription factor 1	3.76	3.63E‐23	3.74	7.42E‐21
*MYO1A*	Myosin IA	3.83	9.30E‐15	3.28	1.73E‐05
*CDC25C*	Cell division cycle 25C	3.9	5.37E‐24	3.84	4.30E‐20
*GPC3*	Glypican 3	4.28	2.79E‐12	3.38	1.91E‐08
*CSMD1*	CUB and Sushi multiple domains 1	5.26	2.78E‐12	4.25	1.23E‐07
*SIX1*	SIX homeobox 1	5.27	1.52E‐12	4.59	2.09E‐18
*IGF2BP1*	Insulin like growth factor 2 mRNA binding protein 1	6.34	3.80E‐28	4.46	1.11E‐08
*ZIC2*	Zic family member 2	6.56	6.91E‐22	5.64	1.03E‐14

Abbreviations: log_2_FC, log_2_ fold‐change; FDR, false discovery rate.

### 3.4 Biomarker identification and confirmation

We used RandomForest (Liaw & Wiener, 2002), a decision tree‐based classification method, to identify biomarkers. A decision tree uses a tree‐like graph, which each branch represents a “test” on an attribute (e.g., whether a gene turned on or not, or if the expression level >20), and each leaf node represents the outcome of the test, usually it is a class label (e.g., “Yes” or “No,” “tumor” or “nontumor”). RandomForest builds a forest of decision trees to make classifications and rank the importance of attributes (e.g., genes).

In our analysis, we splitted the 40 patients data into two data sets. One set was the training data to train the decision tree; the other used as validation data. We used 80% of original data as training data, after training, we used the trees to predict the patient's condition (normal, recurrent tumor, or nonrecurrent tumor) in the validation data set (20% of original data) and compared the prediction with the patient's real condition. If the accuracy was over 80%, we kept the trees and listed the top 30 important genes in the trees according to the importance plot (data not shown). The top 30 genes for reference and de novo assembly approaches are listed in Table 2.

**Table 2:** Top 30 biomarkers that are used to predict recurrence

Reference			De novo assembly		
	Name	Description		Name	Description
**1**	***PLP1***	**Proteolipid protein 1**	**1**	***MT1JP***	**Metallothionein 1J**
2	*MT1M*	Metallothionein 1M	**2**	***SYT9***	**Synaptotagmin 9**
**3**	***SYT9***	**Synaptotagmin 9**	3	*TH*	Tyrosine hydroxylase
4	NA	LincRNA	4	*MT1F*	Metallothionein 1F
5	*KRT16P2*	Keratin 16 pseudogene 2	**5**	***CYP1A2***	**Cytochrome P450 family 1 subfamily A member 2**
6	*KRT16P1*	Keratin 16 pseudogene 1	6	*CLEC4M*	C‐type lectin domain family 4 member M
**7**	***MT1JP***	**Metallothionein 1J**	7	*PLIN2*	Perilipin 2
8	*KRT16P3*	Keratin 16 pseudogene 3	**8**	***HAMP***	**Hepcidin antimicrobial peptide**
9	NA	LincRNA	9	*SYT10*	Synaptotagmin 10
**10**	***CYP1A2***	**Cytochrome P450 family 1 subfamily A member 2**	**10**	***CLEC4G***	**C‐type lectin domain family 4 member G**
11	*ZAN*	Zonadhesin	11	*CD209*	CD209 molecule
12	*CLEC1B*	C‐type lectin domain family 1 member B	12	*RAB25*	RAB25 member RAS oncogene family
**13**	***HAMP***	**Hepcidin antimicrobial peptide**	13	*FAM83F*	Family with sequence similarity 83 member F
**14**	***CLEC4G***	**C‐type lectin domain family 4 member G**	14	*CD5L*	CD5 molecule like
15	*LYPD2*	LY6/PLAUR domain containing 2	**15**	***MARCO***	**Macrophage receptor with collagenous structure**
**16**	***MARCO***	**Macrophage receptor with collagenous structure**	**16**	***MT1G***	**Metallothionein 1G**
17	*MT1X*	Metallothionein 1X	**17**	***PLP1***	**Proteolipid protein 1**
**18**	***MT1G***	**Metallothionein 1G**	18	*MT1E*	Metallothionein 1E
19	*MT1H*	Metallothionein 1H	19	*GDF2*	Growth differentiation factor 2
20	NA	LincRNA	20	*NRDE2*	NRDE necessary for RNA interference domain containing
21	*DHAP8*	Double Homeobox A Pseudogene 8	21	*SLITRK6*	SLIT and NTRK like family member 6
22	*IBSP*	Integrin binding sialoprotein	22	*GPM6A*	Glycoprotein M6A
23	*CLEC2L*	C‐type lectin domain family 2 member L	23	*ADGRA1*	Adhesion G protein‐coupled receptor A1
24	*TOP2A*	Topoisomerase (DNA) II alpha	24	*SFRP5*	Secreted frizzled related protein 5
25	NA	RNA gene	25	*AGBL4*	ATP/GTP binding protein‐like 4
26	*UCHL1*	Ubiquitin C‐terminal hydrolase L1	26	*B3GNT5*	Beta‐1,3‐N‐Acetylglucosaminyltransferase 5
27	*CDCA7*	Cell division cycle associated 7	27	*CKAP2L*	Cytoskeleton associated protein 2 like
28	NA	RNA gene	28	*MCF2L2*	MCF2 cell line derived transforming sequence‐like 2
29	*EXO1*	Exonuclease 1	29	*DUXAP9*	Double homeobox A pseudogene 9
30	NA	RNA gene	30	*TLX1*	T‐cell leukemia homeobox 1

Note

The biomarkers identified by both methods were in bold.

Then we used these top 30 genes (Table 2) to predict the patient condition in the confirmation data set, which had 50 HCC patients’ tumor and normal samples. With these 30 biomarkers, the accuracy for predicting the normal and tumor condition was 100% and 98%, respectively, suggesting these genes might be used for potential biomarkers to predict HCC.

Interestingly, we identified a group of Metallothionein genes as biomarkers (down‐regulated in tumor samples), including metallothionein 1E,1F,1G,1H,1J,1M,1X (Table 3). Metallothioneins (*MT*), are a groups of cysteine‐rich, low molecular weight proteins that bind to heavy metals. Their major function is protection against DNA damage, oxidative stress, and apoptosis, and they play an important role in transcription factor regulation. Therefore, defects in *MT* expression may lead to malignant transformation of cells and ultimately cancer. It has previously been reported that metallothionein is associated with tumors (Arriaga, Bravo, Mordoh, & Bianchini, 2017; Cherian, Jayasurya, & Bay, 2003; Han et al., 2013; Zheng et al., 2017). Here, *MT* were all down‐regulated in tumor samples, suggesting that low *MT* expression may be a promoter of tumor growth.

**Table 3:** Biomarker‐Metallothionein expression and significance level identified using references and de novo assembly

	Name	log_2_FC	FDR
Genes from reference			
ENSG00000205364	metallothionein 1M	−5.33	9.04E‐34
ENSG00000255986	metallothionein 1J	−4.72	5.95E‐26
ENSG00000125144	metallothionein 1G	−4.10	3.96E‐18
ENSG00000187193	metallothionein 1X	−4.10	1.92E‐20
ENSG00000205358	metallothionein 1H	−4.07	2.67E‐13
ENSG00000198417	metallothionein 1F	−3.85	2.24E‐24
ENSG00000169715	metallothionein 1E	−3.57	3.09E‐16
ENSG00000125148	metallothionein 2A	−3.35	4.09E‐17
ENSG00000205361	metallothionein 1D	−3.13	3.46E‐08
ENSG00000260549	metallothionein 1L	−3.10	5.87E‐19
Genes from trinity			
ENSG00000255986	metallothionein 1J	−5.53	4.70E‐26
ENSG00000198417	metallothionein 1F	−4.84	1.47E‐22
ENSG00000125144	metallothionein 1G	−4.15	4.40E‐19
ENSG00000169715	metallothionein 1E	−4.11	3.71E‐16
ENSG00000205358	metallothionein 1H	−3.75	3.25E‐12
ENSG00000260549	metallothionein 1L	−3.59	9.01E‐17
ENSG00000205361	metallothionein 1D	−3.24	9.65E‐15

Abbreviations: log_2_FC, log_2_ fold‐change; FDR, false discovery rate.

## 4 DISCUSSION

In this research, we used both the Reference and de novo assembly approaches to identify genes that could be used as biomarkers to predict recurrence in HCC. We analyzed one RNA‐Seq dataset that with the recurrent tumors after orthotopic liver transplantation (and their paired normal samples) and the nonrecurrent tumor after orthotopic liver transplantation (and their paired normal samples). We did both de novo transcriptome assembly and reference‐based analysis because through our previous research, we discovered that de novo assembly is valuable even when a reference genome available (S. Wang & Gribskov, 2017). And we indeed identified some unique and interesting biomarkers that were not showed in reference method. For example, *CLEC4M*, a protein encodes a transmembrane receptor and expressed in the endothelial cells of the lymph nodes and liver, together with *CD209*, mediate transinfection of liver cells by HCV (Cormier et al., 2004). Another example is *PLIN2*, belonging to the perilipin family, members of intracellular lipid storage droplets. This protein is found in hepatocytes in alcoholic liver cirrhosis, suggesting that it may serve as a marker of lipid accumulation in liver diseases (Graffmann, Ring, Kawala, Wruck, & Ncube, 2016). And *CD5L*, a key regulator of lipid synthesis, was also identified as a possible marker in liver disease (Gangadharan, Antrobus, Dwek, & Zitzmann, 2007). Therefore, these results further confirmed the necessity of conducting a de novo assembly.

In addition to some unique biomarkers identified in de novo method, it was also very interesting that we identified some long noncoding RNA in reference method. Since long noncoding RNA plays important roles in regulating gene expression, these long‐noncoding RNA may be promising biomarkers in HCC diagnostics. However, because we used BLAST program to match the assembled Trinity transcripts to known cDNA gene file, long noncoding RNA was not in the cDNA gene file, therefore, no long noncoding RNA identified in de novo assembly. Furthermore, lower expression of metallthionein protein in HCC tumor has been found before (Cherian et al., 2003), but through our analysis, we systematically pointed out that these genes may use as biomarkers in HCC. However, we recommend more analyses and molecular experiments are needed to confirm the utility of these biomarkers.

In terms of de novo transcriptome assembly programs, it was suggested that SOAPdenovo‐trans and Trinity were the best in case of Arabidopsis study (S. Wang & Gribskov, 2017). In this study, we used Trinity, but we found that Trinity produced many redundant or duplicated transcripts when compared with human reference gene annotation. Therefore it may be advantageous using more transcriptome assembly programs in de novo assembly. And for the bioinformatics analysis, DEG were usually discovered by comparing two conditions at one time. But in our analysis, we compared four conditions simultaneously, taking into account the group (recurrent or nonrecurrent) and condition (normal or tumor) information into the integrated statistical model, therefore improves the accuracy of identifying the significant DEG.

## CONFLICT OF INTEREST

The authors declare no conflict of interest.
